# List of Physicians, Sufferers by the Fire

**Published:** 1871

**Authors:** 


### LIST OF PHYSICIANS

***Sufferers by the late Fire, October 8th and 9th, 1871.***

The following list comprises the names and former and present offices and residences of physicians who have been burned out, corrected up to Nov. 15, as far as it was possible to ascertain.

1. Adam, A.—Residence, 438 N. Wells st.; office, 226 N. Clark st. All within the burnt district. Loss, total. Present location, S. Halsted st. and Canalport av.
2. Acharius, W.—Residence, 130 Townsend st.; office, 49 Chicago av. All within burnt district. Loss, total. Present location, 162 W. Harrison street.
3. Arndt, P. S.—Residence and office, 154 Madison. All in burnt district. Loss, total. Present location, 95 W. Randolph st.
4. Ashworth, Charles.—Residence and office; all in burnt district. Loss total.
5. Avery, S. J.—Residence and office, 154 Madison st.; all in burnt district. Loss, total. Present residence, 328 Walnut st.; present office, 95 W. Randolph street.

6. Baxter, A. J.—Residence, 263 Ontario st.; office, 121 S. Clark; all within the burnt district. Loss, total. Present residence, 185 S. Morgan; present office, 49 S. Halsted.
7. Buckley, C. C.—Residence, 287 Illinois st.; office, 37 Rush st.; all in the burnt district. Loss, total. Present residence, 345 W. Randolph st.
8. Blaney, J. V. Z.—Residence, 289 Illinois st.; office and laboratory, 143 Madison; all in the burnt district. Loss, total. Present residence, 1025 Wabash av.; present office, cor. State and 18th sts.
9. Barnes, Norman S.—Residence and office, cor. State and Madison; in the burnt district. Loss, total.
10. Barnes, P. S.—Residence and office, 66 Madison; in burnt district. Loss, total. Present residence and office, 328 W. Madison st.
11. Bogue, R. G.—Residence, 9 Washington place; office, 100 Washington st.; in the burnt district. Loss, total. Present office and residence, 321 W. Monroe st.
12. Blanchard, Wallace.—Office, 81 Monroe st.; in burnt district. Loss, contents of above. Present residence and office, 265 Michigan av.
13. Brooks, J. W.—Office, 55 Clark st.; in the burnt district. Loss, contents of office. Present residence and office, 857 Wabash av.
14. Bond, T. S.—Office, 47 S. Clark; in the burnt district. Loss, contents of office. Present residence and office, 16 Centre av.
15. Byford, W. H.—Residence, 194 Michigan av.; office, 62 State; all in the burnt district. Loss, nearly total. Present residence, Cottage Grove av.; present office, 785 Wabash av.
16. Case, L. W.—Residence, 317 Division st.; office, Rush Medical College; in the burnt district. Loss, total, except books, clothing and bedding. Present residence, Newberry School.
17. Cunningham, George P.—Residence and office, 129 Oak st.; in the burnt district. Loss, total. Present residence, cor. Jefferson and Randolph streets.
18. Croskey, William.—Residence and office, 98 3rd av.; in the burnt district. Loss, total. Present residence, 913 Indiana av.
19. Carleman, M. B.—Residence, 76 Sedgwick st.; in burnt district. Loss, total, except some bedding and clothing. Present residence, 48 4th street.
20. Cooley, Orrin.—Residence, 374 Chicago av.; office, 370 Chicago av.; in the burnt district. Loss, total. Present residence and office, 260 S. Halsted street.
21. Dysart, J. W.—Residence and office, 447 N. Clark st.; in burnt district. Loss, total. Present residence, 289 W. Randolph st.
22. Freer, J. W.—Residence, 224 Ontario st.; office, Rush Medical College; in burnt district. Loss, total. Present residence and office, 1045 Wabash avenue.
23. Fiske, Calom P.—Residence and office, 249 S. Clark st.; in the burnt district. Loss, total. Present residence, 14 Willard place.

24. Geiger, Henry.—Residence and office, 250 N. Clark st.; in burnt district. Loss, total. Present residence, 39 Archer av.
25. Gray, A. W.—Residence and office, 66 Madison st.; in the burnt district. Loss, total. Present residence, 667 Fulton st.
26. Garvin, H. D.—Office, 169 Dearborn st.; in burnt district. Loss, contents of above. Present office, 255 W. Madison st.
27. Hurlbut, V. L.—Residence and office, 104 Randolph st. Loss, contents of same. Present office, 364 Wabash av.
28. Herz, Cornelius.—Residence, 630 N. La Salle st.; in burnt district. Loss, total. Present residence, 114 S. Jefferson st.
29. Hyde, James N.—Residence and office, 101 S. Clark st.; in burnt district. Loss, total. Present office, 125 22nd st.
30. Hempstead, Charles M.—Office, 55 S. Clark st.; in burnt district. Loss, contents of office. Present residence and office, Ashland av.
31. Hahn, James A.—Office, cor. Dearborn and Randolph sts.; in the burnt district. Loss, contents of office. Present residence and office, 430 Michigan av.
32. Hunt, W. C.—Residence, 271 Erie st.; office, Rush Medical College; in burnt district. Loss, total. Present residence, 657 Monroe st.; present office, 318 W. Madison st.
33. Hollister, J. H.—Office, 164 State st.; in burnt district. Loss, contents of office. Present office, cor. State and 18th sts.
34. Kelly, F. W.—Residence, 250 Wabash av.; office, 154 Washington st.; in burnt district. Loss, total. Present residence, 96 W. Washington st.
35. Knox, W. A.—Office, 106 S. Clark st.; in burnt district. Loss, contents of above. Present residence, 535 W. Adams st.; present office, 185 W. Madison street.
36. Kirschstein, H.—Residence, 252 5th av.; office, 249 S. Clark; in burnt district. Loss, total. Present residence, 194 Cottage Grove av.; present office, 394 S. Clark st.
37. Leonard, R. L.—Residence, 179 Huron st.; office in Mariner's Chapel; in burnt district. Lost, almost total. Present residence, 139 N. Morgan street.
38. Lyman, W. C.—Office, 119 S. Clark st.; in burnt district. Loss, contents of above.
39. Loverin, N.—Residence and office, 208 Randolph st.; in burnt district. Loss, total. Present residence, 146 W. Jackson st.
40. Leslie, S.—Residence and office, 190 S. Clark st.; in burnt district. Loss total, excepting $200 of clothes and instruments. Present residence, 387 State street.
41. McDonald, P. S.—Office, cor. Clark and Harrison sts.; in burnt district. Loss, office and contents. Present office, 351 S. Clark st.
42. McArthur, J. D.—Residence and office, 234 Dearborn st.; in burnt district. Loss, total. Present office, 50 W. Madison st.
43. Mackey, H. A.—Residence, 272 Indiana st.; in burnt district. Loss, total. Present residence, 760 State st.

44. Morgan, A. G.—Residence, 101 Huron st.; in burnt district. Loss, total, except a little furniture in damaged condition. Present residence, 246 Fulton st.

45. Martin, William.—Residence and office, 112 Randolph; in burnt district. Loss, total. Present residence and office, 589 Wabash av.

46. Maynard, Wm. J.—Residence at Marine Hospital; office, 21 Washington st.; in burnt district. Loss, total. Present residence and office, 424 W. Van Buren street.

47. Merriman, H. P.—Office, cor. State and Monroe; in burnt district. Loss, contents of above. Present office, 785 Wabash av.

48. O'Ryan, Charles D. B.—Residence, 248 N. Wells st.; in burnt district. Loss, total. Present residence, Jefferson, Cook Co.

49. Parkes, Charles T.—Residence, 224 Ontario st.; office, Rush Medical College; in burnt district. Loss, total. Present residence, 462 Elston av.; present office, 121 22nd st.

50. Powell, Edwin.—Residence and office, 45 S. Clark st.; in burnt district. Loss total. Present office, 318 W. Madison st.

51. Price, O. J.—Residence, 65 W. Van Buren st.; in burnt district. Loss, total. Present residence, 92 W. Van Buren st.

52. Reed, John.—Residence, 15 Washington place; burnt district, North side. Loss, total. Present office, 273 W. Randolph st.

53. Simpson, John.—Residence, No. 6 W. Oak st.; in burnt district. Loss, total. Present residence, 166 Curtis st.

54. Smith, H. R.—Residence, 167 S. Clark st.; in burnt district. Loss, total. Present residence, 1100 Indiana av.

55. Scheppers, D. Q.—Residence, 292 Larrabee st.; in burnt district. Loss, total. Present residence, 157 Mather st.

56. Savage, A. C.—Residence and office, 233 Madison st.; in burnt district. Loss, total. Present residence, 133 W. Madison st.

57. Schwarz, H.—Residence, 184½ S. Clark st.; office, 41 N. Clark st.; in burnt district. Loss total. Present residence, 62 Milwaukee av.

58. Sherman, Julien S.—Residence, 297 Wabash av.; office, 81 Monroe st.; in burnt disrict. Loss, total.

59. Starkweather, Ralph E.—Office, 169 Dearborn st.; in burnt district. Loss, contents of above. Residence and office, 368 Michigan av.

60. Smith, Charles G.—Residence and office, 169 Dearborn st.; in burnt district. Loss, total. Present office, 100 16th st. and 229 W. Madison.

61. Thibodo, R.—Residence and office, 41 N. Clark street; in burnt district, Loss, total. Present residence, 125 N. Desplaines st.

62. Thompson, D. D.—Office, 169 Dearborn st.; in burnt district. Loss, office and contents. Present and former residence, 37 Myrick av.

63. Teare, John.—Residence, 129 Huron st.; in burnt district. Loss, total. Present residence, 152 N. Sangamon st.

64. Trimble, D. B.—Residence, Geneva, Wisconsin; office, 156 N. Clark st.; office in burnt district. Loss, a few books. Present office, 119 22nd st.

65. Varges, L.—Residence and office, 680 Sedgwick st.; in burnt district. Loss total. Present residence, 159 Cottage Grove av.
66. Van Doozer, B. R.—Residence, 122 3rd av.; office, 112 oMnroe st.; in burnt district. Loss, total. Present residence, 420 Burnside st.; present office, 120 S. Halsted st.
67. Weeks, J. F.—Residence and office, No. 118 S. Clark st.; in burnt district. Loss, total.
68. Wagner, William.—Residence 363 N. Clark st.; office, 28 N. Clark st.; in burnt district. Loss, total. Present residence, 295 W. Lake st.
69. Williams, J. F.—Residence, 121 Lincoln av.; office, 156 N. Clark st.; in burnt district. Loss, total. Present residence, 62 Grant place.
70. Ware, Lyman—Office, 169 Dearborn st.; in burnt district. Loss, content of office, library and instruments. Present office, 388 State st.
71. Young, Fern—Residence and office, 184 S Clark st.; in burnt district. Loss, total. Present residence, 204 W. Madison.

